# Dotinurad Treatment for Patients With Hyperuricemia Complicating CKD

**DOI:** 10.1016/j.ekir.2025.03.047

**Published:** 2025-04-03

**Authors:** Katsuyuki Tanabe, Tomokazu Nunoue, Naoki Itabashi, Akihiro Katayama, Akihiko Nakamura, Hiroyuki Ohbayashi, Yasuhiro Onishi, Kyoko Watanabe, Keisuke Maruyama, Takeshi Hosoya, Shinichi Okada, Jun Wada, DTN-CKD Investigators

**Affiliations:** 1 Department of Nephrology, Rheumatology, Endocrinology and Metabolism, Okayama University Graduate School of Medicine, Dentistry, and Pharmaceutical Sciences, Okayama, Japan; 2 Nunoue Clinic, Tsuyama, Japan; 3 Itabashi Diabetes and Dermatology Medical Clinic, Ibaraki, Japan; 4 NHO Okayama Medical Center, Okayama, Japan; 5 Osafune Clinic, Setouchi, Japan; 6 Tohno Chuo Clinic, Gifu, Japan; 7 Japanese Red Cross Okayama Hospital, Okayama, Japan; 8 Okayama Saiseikai Outpatient Center Hospital, Okayama, Japan; 9 Hosoya Clinic, Ibara, Japan; 10 Okada Medical Clinic, Okayama, Japan

**Keywords:** chronic kidney disease, dotinurad, efficacy, hyperuricemia, safety, serum uric acid

## Abstract

**Introduction:**

The management of hyperuricemia is important to reduce cardiovascular risk and the progression of renal injury in chronic kidney disease (CKD). This study aimed to assess the efficacy and safety of dotinurad, a novel urate transporter-1 inhibitor, in patients with hyperuricemia and CKD.

**Methods:**

In a nonrandomized, parallel interventional study, patients were grouped based on their estimated glomerular filtration rate (eGFR) at baseline. The starting dotinurad dose was 0.5 mg/d and titrated to a final dose of 2 mg/d to 4 mg/d. The primary end point was the noninferiority of the change in serum uric acid (UA) levels between the G1/G2 and G3/G4 groups at week 24. The main secondary end points were changes in eGFR and UA clearance-to-creatinine clearance ratio (C_UA_/C_Cr_). Reported adverse events were also investigated.

**Results:**

Ninety-eight patients continued the dose titration. The mean percentage reduction in serum UA level at week 24 were 47.2% and 42.8% for the G1/G2 and G3/G4 groups, respectively; the between-group difference was −4.3% (95% confidence interval [CI], −9.5% to 0.9%, noninferiority *P* = 0.0321), validating the noninferiority of treatment in the G3/G4 group to the G1/G2 group. eGFR tended to increase slightly through to week 24, suggesting that spontaneous eGFR decline was counteracted. Mean C_UA_/C_Cr_ generally increased over time from week 4 to week 24. No new safety issues of particular concern were identified; and there were no marked changes in urinary pH.

**Conclusion:**

Dotinurad therapy may be well-tolerated in patients with hyperuricemia and may have efficacy comparable with existing standard treatment in patients with CKD stages G3/G4. Randomized controlled trials in larger patient groups are needed.

## Graphical abstract

**Figure:**
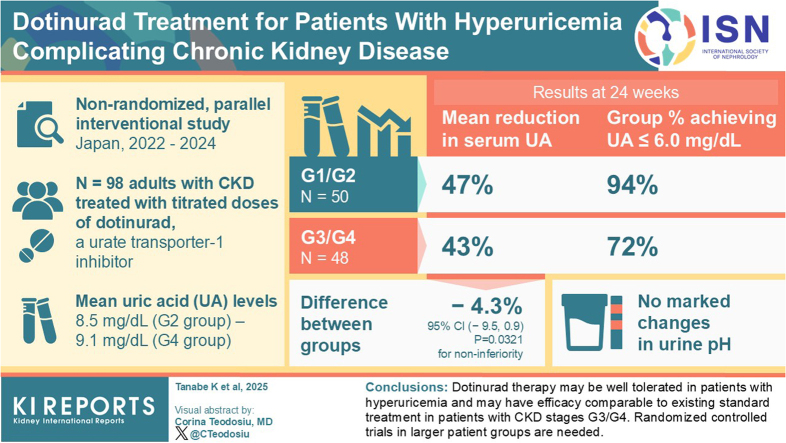

See Commentary on Page 1630

Hyperuricemia and gout are frequently associated with CKD.^1^ A meta-analysis reported a positive association between serum UA level and CKD, independent of established metabolic risk factors, including diabetes and hypertension, with a relative risk of CKD of 1.22 per 1 mg/dl increment in serum UA level.^2^ The relationship between CKD and UA may be bidirectional; UA has recently drawn attention as a potential modifiable risk factor in the development and progression of CKD.^3^

Evidence-based urate-lowering drug therapies (ULTs) for hyperuricemia associated with CKD include allopurinol and other urate production inhibitors.^4^^,^^5^ The American College of Rheumatology guidelines recommend allopurinol as the preferred first-line treatment for gout, including in patients with CKD stages ≥ 3.^4^ Japanese guidelines also recommend the use of urate production inhibitors according to their benefit–risk balance.^5^ In contrast, uricosuric drugs that promote UA excretion are not recommended in patients with impaired renal function because they may be less effective as renal function deteriorates. It has also been thought that there may be a risk of UA stone and crystal formation in the urinary tract. However, UA excretion is not necessarily impaired in the setting of CKD, and treatment with uricosuric drugs may be effective.^6^ Among uricosuric drugs, benzbromarone has been reported to be effective in CKD-associated hyperuricemia, including in CKD stages ≥ G4 (eGFR < 30 ml/min per 1.73 m^2^) as monotherapy or in combination with allopurinol.^7^, ^8^, ^9^, ^10^ Given that fewer patients with hyperuricemia and reduced eGFR in CKD stages G3 to G5 reach target UA levels compared with those with normal renal function, further treatment options, in addition to the available urate production inhibitors, are desirable in such patients.^11^ To the best of our knowledge, the present study is the first prospective exploratory interventional study to examine the hypothesis that dotinurad therapy might offer a beneficial treatment option in patients with hyperuricemia and CKD stages G3/G4.

Dotinurad is a novel uricosuric drug, which is characterized as a selective urate reabsorption inhibitor that acts via selective inhibition of the renal urate transporter UA transporter-1, promoting the excretion of UA.^12^ It has been evaluated for noninferiority to benzbromarone and febuxostat as well as for safety and efficacy in long-term use.^13^, ^14^, ^15^ In a single-dose pharmacokinetic study of dotinurad in patients with CKD stages G2/G3, serum UA reduction was attenuated in those with CKD stage G3 compared with patients with CKD stage G1.^16^ Thus, a precautionary statement was established for dotinurad use in patients with severe renal impairment (eGFR < 30 ml/min per 1.73 m^2^).^17^ In contrast, an integrated analysis of clinical trials showed no clear trend toward attenuated changes in serum UA levels for patients with CKD stage G3, and no safety issues were identified.^18^ However, the number of patients with CKD stages G3a and G3b in clinical trials to date is small.^18^ Only 25 patients with CKD stage G3b have been evaluated across 5 clinical trials, so the efficacy and safety of dotinurad in patients with CKD stages G3, especially G3b, and G4, require clarification. Such information may lead to better management of UA levels in patients with CKD, including those who cannot receive adequate doses of ULTs because of impaired renal function and those for whom the efficacy of ULTs is insufficient.

The main objective of the current study was to evaluate the efficacy and safety of dotinurad treatment in patients with hyperuricemia with CKD stages G1 to G4 using changes in serum UA levels. Changes in eGFR at 24 weeks and in C_UA_/C_Cr_, urine protein-to-creatinine ratio, and urine albumin-to-creatinine ratio were also assessed.

## Methods

### Study Design

This was a nonrandomized, prospective, parallel interventional study with a 24-week treatment and observation period conducted from February 2022 to March 2024 at 23 sites in Japan. Based on week 0 eGFR values, patients were classified into G1 (eGFR ≥ 90 ml/min per 1.73 m^2^), G2 (eGFR, 60–89 ml/min per 1.73 m^2^), G3a (eGFR, 45–59 ml/min per 1.73 m^2^), G3b (eGFR, 30–44 ml/min per 1.73 m^2^), or G4 (eGFR, 15–29 ml/min per 1.73 m^2^) groups as described in the Japanese Society of Nephrology and the Kidney Disease: Improving Global Outcomes Clinical Practice Guidelines.^19^^,^^20^

### Ethical Approval and Informed Consent

The study was approved by the Okayama University Clinical Research Review Board (Approval No. CRB6180001). It was conducted with the ethical principles based on the Declaration of Helsinki and in compliance with local laws. All patients provided written informed consent. The trial is registered with the Japan Registry of Clinical Trials (jRCT; https://jrct.niph.go.jp/latest-detail/jRCTs061210079).

### Eligibility Criteria

Patients with hyperuricemia aged ≥ 20 years, not treated with ULTs within 2 weeks before study treatment, and who had a serum UA level (untreated) ≥ 8.0 mg/dl and eGFR ≥ 15 ml/min per 1.73 m^2^ within 6 weeks before study treatment were included. Patients with acute gouty arthritis, urinary tract stones, secondary hyperuricemia (for example, to tumor lysis syndrome, aspartate aminotransferase or alanine aminotransferase ≥ 100 IU/L), and pregnant or lactating women were excluded.

### Intervention or Exposure

The dotinurad starting dose was 0.5 mg once daily, titrated to a final dose of 2 mg/d (maintenance dose) to 4 mg/d. The standard titration schedule was as follows: week 0, 0.5 mg/d; week 4, 1 mg/d; and week 8, 2 mg/d. If the serum UA level during the 2 mg/d administration did not reach the target UA level (≤ 6.0 mg/dl), the dose was increased up to a maximum of 4 mg/d. The timing of the dose increase could be changed or delayed, depending on any complications or adverse events.

The concomitant use of ULTs other than dotinurad was prohibited. Concomitant use restrictions were placed on angiotensin-converting enzyme inhibitors, angiotensin II receptor blockers, thiazide diuretics, loop diuretics, and sodium-glucose cotransporter 2 inhibitors, which were to be continued unchanged if used at the time of initiation of dotinurad and not newly started except, for example, in cases of adverse event development.

### Study End Points

The primary end point was to verify the noninferiority of the change in serum UA levels between the G1/G2 and G3/G4 groups at week 24. The relative and absolute change from baseline in serum UA level at each evaluation time point, and percentage of patients reaching serum UA ≤ 6.0 mg/dl, set as the target UA level in this study, were calculated for the G1/G2 and G3/G4 groups and the G1, G2, G3a, G3b, and G4 groups. Serum UA ≤ 6.0 mg/dl has been supported by Japanese guideline as a management target UA level for patients with hyperuricemia receiving ULT who have comorbid conditions^5^; it has also been employed in other clinical trials as a target level for ULT efficacy assessment in patients with hyperuricemia.^12^, ^13^, ^14^, ^15^

Secondary end points were the percentage change and change in eGFR at 24 weeks for the G1/G2 and G3/G4 groups and the G1, G2, G3a, G3b, and G4 groups, and changes in C_UA_/C_Cr_, urine protein-to-creatinine ratio, and urine albumin-to-creatinine ratio.

### Safety

Data on adverse events that occurred after the start of dotinurad administration were collected. The number and percentage of patients who had adverse drug reactions with a possible causal relationship with dotinurad were evaluated. Descriptive statistics were calculated for test values and their changes from week 0 of liver function tests (aspartate aminotransferase, alanine aminotransferase, and γ-glutamyltranspeptidase) and urinary pH in the G1/G2 and G3/G4 groups, as well as the G1, G2, G3a, G3b, and G4 groups at each evaluation time point. Adverse events were coded using the Medical Dictionary for Regulatory Activities Version 26.0.

### Sample Size Calculation

A mean of 45% in the rate of change in serum UA levels at the end of treatment was predicted based on phase 3 studies.^13^^,^^14^ The rate of change was assumed to be similar in the G1/G2 and G3/G4 groups (0% difference between groups), with an SD of 15%. The number of patients required to validate the noninferiority of the G3/G4 group to the G1/G2 group (−10% noninferiority margin) at 80% power and 2.5% 1-sided significance level was calculated to be 37 patients per group. Considering patients who were not eligible and dropouts, the number of patients per group was set at 50.

### Statistical Analysis

For the primary end point, the rate of change in serum UA level was calculated as follows: (%) = (serum UA at week 0 − serum UA at week 24 or discontinuation) ÷ serum UA value at week 0 × 100. Analysis of covariance was used to calculate the least squares (LS) mean for the rate of change per group with the value at week 0 as the covariate and the point estimate and 2-sided 95% CI for the difference between groups. The significance level was set at 2.5% 1-sided, and the lower limit of the 2-sided 95% CI for the between-group difference was considered as verification of noninferiority when it exceeded −10%, which was set as the noninferiority margin. The noninferiority margin of −10% is approximately one-fifth of the difference in UA change obtained with placebo and dotinurad; this margin was used in phase 3 studies.^13^^,^^14^ Point estimates and 2-sided 95% CIs of the percentage of patients reaching the target UA level at each assessment time point for the G1/G2 group and G3/G4 group and the difference between groups (G3/G4 group − G1/G2 group) were calculated. For the primary evaluation of change in serum UA, missing week 24 values were imputed using the last observation carried forward method; other missing values were not imputed.

For the secondary end points, the percentage change in eGFR was calculated as follows: (%) = (eGFR value at week 0 − eGFR value at week 24 or discontinuation) ÷ eGFR value at week 0 × 100%. Point and 95% CI estimates of the LS mean were calculated for the percent change and change in eGFR for the G1/G2 group, G3/G4 group, as well as the G1, G2, G3a, G3b, and G4 groups. Summary statistics were calculated for the measured values and the change from week 0 for urine protein-to-creatinine ratio, urine albumin-to-creatinine ratio, and C_UA_/C_Cr_. Correlation analyses were performed to assess the relationship between the serum UA level change and eGFR change at week 24. The efficacy analysis was performed on the per protocol set, and safety analysis was performed on the safety analysis population. All statistical analyses were performed using SAS software (SAS for Windows, SAS Institute Inc).

## Results

### Patient Flow Diagram

**Figure 1:**
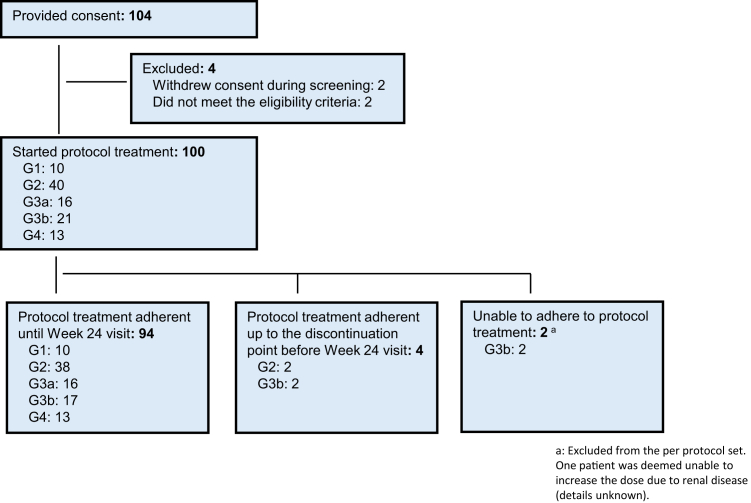
Participant flow diagram.

**Figure 2:**
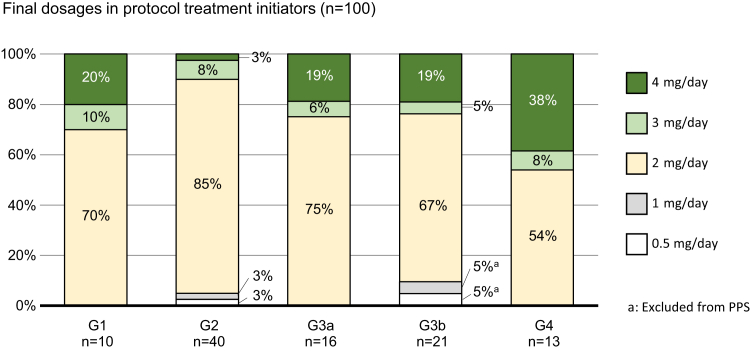
Dotinurad final dosages. Some percentage values do not total 100% because of rounding of numbers. PPS, per protocol set.

Consent was obtained from 104 patients (Figure 1). After excluding 4 patients who had not received the study drug (2 withdrew consent and 2 were ineligible), treatment was initiated in 100 patients, and all were included in the safety analysis. Of those, 2 discontinued study treatment because of intolerance. Ninety-eight patients continued the prescribed titration and all were included in the per protocol set; 4 patients discontinued; 94 patients completed 24 weeks of treatment and reached the maximum dose of 2 mg/d. Final dosages were as follows: 0.5 mg/d, n=1; 1 mg/d, n=1 (reduced from 2 mg/d); 2 mg/d, n=74; 3 mg/d, n=7; and 4 mg/d, n=15. A relatively high percentage of patients with CKD stages G3a, G3b, and G4 had final doses > 2 mg/d (Figure 2).

### Patient Background

In Table 1 and Supplementary Table S1, we present the patient background. Age tended to increase and body mass index tended to decrease in the later CKD stages. However, the duration of hyperuricemia and the presence of comorbidities were comparable among groups; mean serum UA levels at week 0 were comparable among the G1–G4 groups, ranging from 8.5 mg/dl (G2 group) to 9.1 mg/dl (G4 group) (Supplementary Table S2). No patients deviated from the criteria for use of concomitant medications by newly initiating or increasing the dose of restricted concomitant medications during the study period.

**Table 1:** Patient background by G1/G2 and G3/G4 group

Group	G1/G2 (*n* = 50)	G3/G4 (*n* = 48)
Sex		
Male	47 (94)	39 (81)
Age (yrs)		
Mean (SD)	51 (14)	65 (13)
20–59	36 (72)	15 (31)
≥60	14 (28)	33 (69)
BMI (kg/m^2^)		
Mean (SD)	28.3 (4.7)	25.5 (4.3)
18.5 to <25	15 (30)	27 (56)
25 to <30	16 (32)	14 (29)
≥30	19 (38)	7 (15)
Duration of hyperuricemia (yrs)		
Mean (SD)	2.9 (4.7)	4.2 (7.0)
<1	25 (50)	26 (54)
1–9	19 (38)	14 (29)
≥10	6 (12)	8 (17)
Serum UA level (mg/dl)		
Mean (SD)	8.6 (0.9)	8.9 (0.9)
Min, max	4.8, 10.1	7.7, 12.6
eGFR (ml/min per 1.73 m^2^)		
Mean (SD)	78.1 (14.5)	38.0 (12.2)
Min, max	60.3, 130.3	15.8, 58.5
Urine protein-to-creatinine ratio (g/gCr)		
Mean (SD)	0.144 (0.303)	0.964 (1.402)
Min, max	0.01, 1.84	0.02, 5.39
Urine albumin-to-creatinine ratio (mg/gCr)		
Mean (SD)	79.8 (233.4)	666.1 (947.4)
Min, max	3, 1248	4, 3230
History of gout		
Yes	10 (20)	7 (15)
CKD^a^		
Yes	0	48 (100)
Causal disease		
Diabetic nephropathy	0	14 (29)
Nephrosclerosis	0	18 (38)
IgA nephropathy	0	5 (10)
Membranous nephropathy	0	1 (2)
Focal glomerulosclerosis	0	2 (4)
Polycystic kidney disease	0	2 (4)
Unknown cause	0	3 (6)
Others	0	3 (6)
Malignant neoplasm of renal pelvis	0	1 (2)
Nephrectomy	0	1 (2)
Toxic nephropathy	0	1 (2)
Comorbidities		
Yes	43 (86)	45 (94)
Diabetes	23 (46)	24 (50)
Dyslipidemia	28 (56)	32 (67)
Hypertension	27 (54)	37 (77)
Stroke or CAD	5 (10)	10 (21)
PAD	0	0
Concomitant medications		
Thiazides	4 (8)	2 (4)
Diuretics except thiazides	0 (0)	4 (8)
ARBs	2 (4)	2 (4)
Antihypertensives except diuretics and ARBs	24 (48)	25 (52)
Insulin	1 (2)	8 (17)
SGLT2-inhibitors	15 (30)	16 (33)

ARB, angiotensin II receptor blockers; BMI, body mass index; CAD, coronary artery disease; CKD, chronic kidney disease; eGFR, estimated glomerular filtration rate; IgA, immunoglobulin A; PAD, peripheral artery disease; SGLT2, sodium-glucose cotransporter 2; UA, uric acid.

Values are *n* (%) unless indicated otherwise.

a Defined as eGFR < 60 ml/min per 1.73 m^2^.

### Efficacy

**Figure 3:**
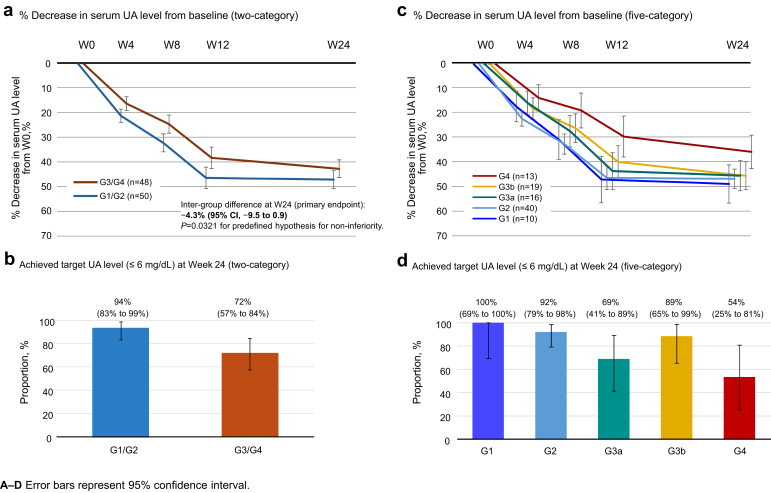
Serum uric acid level. (a) Percentage decrease in serum uric acid level from baseline and (b) percentage of patients achieving target UA level at week 24 in the G1/G2 and G3/G4 groups. (c) Percentage decrease in serum uric acid level from baseline and (d) percentage of patients achieving the target UA level at week 24 in the G1, G2, G3a, G3b, and G4 groups. UA, uric acid; W, week.

Changes in serum UA levels and the percentage of patients reaching the target UA level are shown in Figure 3 and Supplementary Table S2. The percentage change (LS mean) in serum UA level at week 24 was 47.2% (95% CI: 43.6%–50.8%) for the G1/G2 group and 42.8% (95% CI: 39.1%–46.4%) for the G3/G4 group (Supplementary Table S2). The between-group difference by the primary endpoint analysis was −4.3% (95% CI: −9.5% to 0.9%, noninferiority *P* = 0.0321), with a lower limit above −10%, validating the noninferiority of the G3/G4 group to the G1/G2 group (Figure 3a). Overall, 94% of the G1/G2 group and 72% of the G3/G4 group achieved the target UA level at week 24 (Figure 3b). Serum UA level decreased over time from week 4 to week 24 in all groups (G1–G4); the percentage decrease (LS mean) at week 24 was similar among groups, ranging from 45.4% to 48.9%, but lower in the G4 group (35.7%) (Figure 3c, Supplementary Table S2). The percentage of patients reaching the target UA level at week 24 ranged from 54% (G4 group) to 100% (G1 group) (Figure 3d, Supplementary Table S2). In the primary endpoint analysis, imputation using the last observation carried forward method was performed for 2 patients because of missing values.

**Figure 4:**
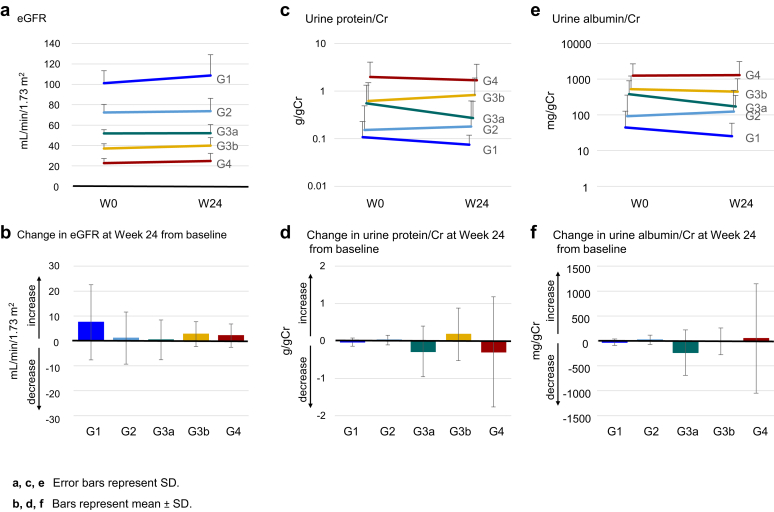
Indicators related to renal function. Changes in the G1, G2, G3a, G3b, and G4 groups in (a and b) eGFR, (c and d) urine protein-to-creatinine ratio, and (e and f) urine albumin-to-creatinine ratio. Cr, creatinine; eGFR, estimated glomerular filtration rate; W, week.

**Figure 5:**
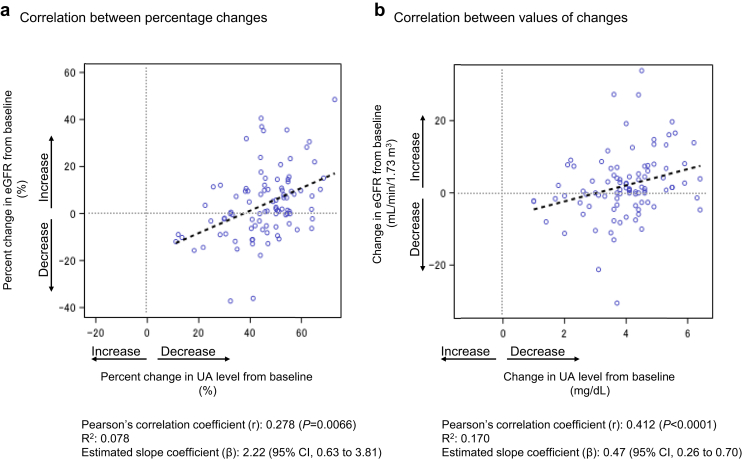
Relationship between changes in serum uric acid levels and changes in eGFR at week 24 from baseline. (a) Correlation between percentage changes; (b) correlation between values of changes. CI, confidence interval; eGFR, estimated glomerular filtration rate; UA, uric acid.

**Figure 6:**
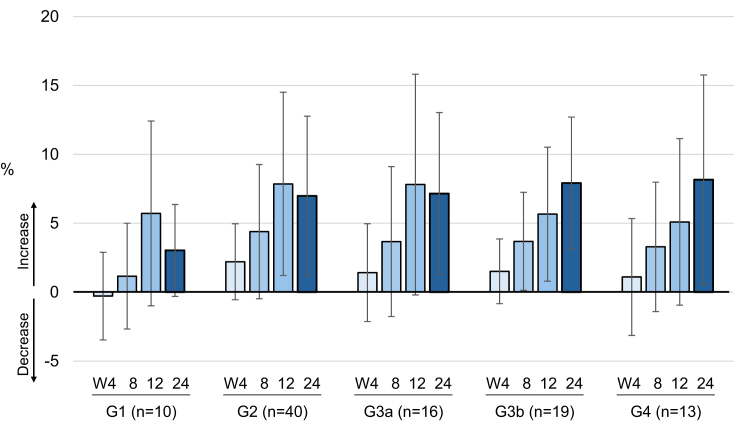
Change (increase) in C_UA_/C_Cr_ (W24 − W0) in the G1, G2, G3a, G3b, and G4 groups. C_UA_/C_Cr_, uric acid clearance-to-creatinine clearance ratio; W, week.

Changes in renal function tests are shown in Figure 4 and Supplementary Table S3. eGFR tended to increase slightly from week 0 to week 24, with the largest change observed in the G1 group (Figure 4a and b). LS mean changes in eGFR in each group at week 24 were as follows: G1, 5.8 (95% CI: −7.4 to 18.9); G2, 0.6 (95% CI: −4.3 to 5.4); G3a, 0.7 (95% CI: −4.3 to 5.7); G3b, 3.5 (95% CI: −4.0 to 11.0); and G4, 3.5 (95% CI: −7.7 to 14.8). This indicated no consistent trend in the magnitude of change, but a positive mean change in all groups (Supplementary Table S3). Changes in urine protein and urine albumin from week 0 to week 24 are shown in Figure 4c–f and Supplementary Table S3. In a *post hoc* analysis to evaluate whether there was any relationship between changes in serum UA levels and changes in urinary protein and albumin at week 24 from baseline, no relationship was observed (Supplementary Figure S1). Correlations between change in serum UA level and change in eGFR at week 24 are shown in Figure 5. Linear regressions were observed for rate and amount of change, with both showing correlation (*R*^*2*^ = 0.078 and *R*^*2*^ = 0.170 for the 2 regression models). Changes in C_UA_/C_Cr_ are shown in Figure 6 and Supplementary Table 4. Mean C_UA_/C_Cr_ generally increased over time from week 4 to week 24; the mean change from baseline at week 24 ranged from 3.0% to 8.2%, with the largest increases observed in the G3b and G4 groups. Of note, whereas the mean changes in C_UA_/C_Cr_ at week 24 were broadly similar for the G3a and G3b groups, the percentage of patients reaching the target UA level (≤ 6.0 mg/dl) at week 24 was 69% for the G3a group and 89% for the G3b group, a difference of 20% (Figure 3d). This difference was incidental, and attributable to slight differences in the distribution of serum UA levels at week 24, particularly in the narrow range of 5–7 mg/dl around the ≤ 6.0 mg/dl target threshold.

### Safety

Adverse drug reactions were reported by 3 patients (8%) in the G2 group (arthralgia, gouty arthritis, and “wound”), and 1 patient (5%) in the G3b group (gout). Adverse drug reactions other than “wound” were known events (Table 2). Regarding changes in liver function (alanine aminotransferase, aspartate aminotransferase, and γ-glutamyl transpeptidase), there was a trend toward a slight increase in liver function parameters at week 24 in the G1 group, but no increase at week 24 in the G3a, G3b, and G4 groups (Supplementary Table 5). Of note, there were no marked changes in urinary pH during the study. At week 24, urinary pH ranged from 5.8 (G3b group) to 6.4 (G4 group). No urinary alkalizers were used at the physician's discretion and no patients had urinary tract stones. The changes in blood glucose and glycated hemoglobin did not indicate clinically notable trends.

**Table 2:** Adverse drug reactions

Adverse drug reactions	Total (*N* = 100)	G1 (*n* = 10)	G2 (*n* = 40)	G3a (*n* = 16)	G3b (*n* = 21)	G4 (*n* = 13)
Any ADR	4 (4)	0	3 (8)	0	1 (5)	0
Gout	1 (1)				1 (5)	
Arthralgia	1 (1)		1 (3)			
Gouty arthritis	1 (1)		1 (3)			
Wound	1 (1)		1 (3)			

ADR, adverse drug reaction.

Values are *n* (%).

## Discussion

This study verified the hypothesis of the noninferiority of serum UA reduction in patients with CKD stage G3/G4 compared with patients with CKD stages G1/G2, which was the primary end point. The mean percentage reduction in serum UA was similar in the G3a (45.4%) and G3b (45.5%) groups versus G1 (48.9%) and G2 (46.8%) groups, and approximately 10% lower in the G4 group (35.7%). The percentage of patients reaching the target UA level was CKD stage-specific, with a lower percentage of the G4 group (54%) achieving the target than the G2 group (92%). The reduction in serum UA in patients in the G3 and G4 groups is generally slightly higher than that observed in studies of febuxostat and allopurinol in patients with CKD stages G3 and G4, which showed reductions in serum UA of approximately 30% to 40% from baseline.^21^, ^22^, ^23^

Regarding safety, except for a “wound,” which was an injury in 1 patient resulting in treatment at another hospital, the adverse effects were known; no adverse effects were observed in the G4 group, and no new safety issues of particular concern were identified. There were no marked changes in urinary pH or liver function during the study. Furthermore, there was no incidence of urinary tract stones with dotinurad titration, including in patients with CKD stage G3 or G4. These findings suggest that dotinurad therapy may be well-tolerated in this patient group, providing a treatment option for managing serum UA levels in patients with CKD stages up to G3/G4.

Additional analysis of data from a phase 3 long-term study of dotinurad^15^^,^^18^ (up to 58-week administration) reported that mean percentage changes in serum UA levels were similar among groups (G1 [*n* = 24, 48.6%], G2 [*n* = 225, 48.9%], G3a [*n* = 61, 47.3%], and G3b [*n* = 9, 47.8%]) in patients treated with dotinurad 2 mg/d to 4 mg/d.^18^ LS mean changes in serum UA in the current study ranged from 45.4% to 48.9% in the G1, G2, G3a, and G3b groups, similar to that observed in the long-term study,^15^ supporting the efficacy results obtained with treatment starting at 0.5 mg/d and increasing to 2 mg/d to 4 mg/d after titration.

Two recent retrospective studies of patients with CKD stages G3b and G4 reported reductions in serum UA of approximately 17% (baseline, 7.1 mg/dl; posttreatment, 5.9 mg/dl)^24^ and 27% (pretreatment, 8.3 mg/ml; posttreatment, 6.1 mg/ml)^25^ with dotinurad treatment. Most patients (approximately 94% and 81%, respectively) in those studies received a final dose of dotinurad < 2 mg/d.^24^^,^^25^ Considering the fact that greater serum UA reductions were observed in our study, where a majority of patients (96%) achieved the final dose of ≥2 mg/d and a high proportion of patients with CKD stage G4 (46%) had their final dose > 2 mg/d, a titrating dose to 2 mg/d or even higher, up to maximum of 4 mg/d, is suggested for enhancing efficacy for patients with CKD stage G3b or G4.

In the current study, positive mean changes in eGFR were demonstrated in all groups (G1–G4), suggesting that decline over time may have been counteracted. This is consistent with previous reports that suggest the possibility of delaying eGFR decline with dotinurad treatment.^18^^,^^24^^,^^25^ The possible renoprotective effect may have been primarily mediated by a reduction in serum UA levels, which is supported by the significant correlation between the changes in serum UA level and eGFR in the current study. Furthermore, the small coefficient of determination (*R*^*2*^) of the linear model reported in our study implies that the change in eGFR with dotinurad treatment may be better explained by a model that considers background factors such as severity of renal dysfunction or concomitant treatment. In addition, factors conferred by a novel mechanism of action of dotinurad, which inhibits UA transporter-1–mediated reabsorption of UA in the proximal tubular cells, may be involved. Dotinurad selectively inhibits UA reabsorption via UA transporter-1 in proximal tubular cells without affecting the secretory pathway of UA via ATP-binding cassette subfamily G member 2, organic anion transporter 1, and organic anion transporter 3 in the intestinal tract.^12^ Verification of whether dotinurad has a favorable effect on renal function decline over time and whether it has a direct mechanism independent of reduction in UA will be a promising topic for further investigation.

In enrolled patients with hyperuricemia in CKD stage G3 or G4, dotinurad titration produced a constant reduction in serum UA levels consistent with that observed in patients with CKD stage G1 or G2, which was supported by the observed changes in C_UA_/C_Cr_. The findings regarding eGFR suggest that dotinurad titration therapy possibly has an inhibitory effect on eGFR decline, with an effect that may involve a mechanism secondary to that of decreased serum UA level.

This study has some limitations, including its nonrandomized design with no placebo comparator and the relatively small sample size, particularly in the G4 group. The study was conducted among only Japanese patients, who were predominantly male; thus, the findings are not necessarily generalizable to settings outside Japan or to female patients. In addition to different baseline eGFR, there were differences among groups in other background characteristics, including age. Therefore, intergroup differences in the magnitude of change may be present in this study. However, comorbidities, duration of hyperuricemia, and mean baseline serum UA levels were generally balanced across the groups. Baseline C_UA_/C_Cr_ levels were slightly higher in the G4 group than in the other groups. This may reflect the higher proportion of the hyperexcretion type in the G4 group, thus introducing a potential selection bias.

In conclusion, the study suggests that dotinurad may become a standard treatment option for the management of serum UA in patients with hyperuricemia in CKD stages G3 and G4. However, to further elucidate use in such patients, longer-term randomized controlled trials that compare dotinurad with standard therapy in larger patient groups are required.

## Appendix

### List of the DTN-CKD Investigators

Kosuke Oota, National Hospital Organization Okayama Medical Center; Shigeru Morioka, Okayama Central Hospital; Naoyuki Gamo, Japanese Red Cross Okayama Hospital; Kosuke Yozai, Okayama Rosai Hospital; Shinji Kamei, Kurashiki Central Hospital; Kenichiro Asano, Kurashiki Central Hospital; Tatuaki Nakatou, Okayama Saiseikai Outpatient Center Hospital; Nobuo Kajitani, Okayama City Hospital; Takahiro Terami, Terami Medical Clinic; Kazuharu Murakami, Murakami Clinic; Masami Hashimoto, Hashimotojin Clinic; Osamu Doi, Kurashiki Riverside Hospital; Yuji Hidaka, Akasaka Central Clinic.

## Disclosure

All the authors received funding support from Fujiyakuhin Co., Ltd. and Mochida Pharmaceutical Co., Ltd. throughout the implementation of the study, including the manuscript writing support. JW received grants from Bayer, Chugai, Kyowa Kirin, Otsuka, Shionogi, Sumitomo, and Mitsubishi Tanabe and speaker honoraria from 10.13039/100004325AstraZeneca, Bayer, Boehringer Ingelheim, Daiichi Sankyo, Kyowa Kirin, Novo Nordisk, and Mitsubishi Tanabe.

## Supplementary Material

Supplementary File (PDF) **Figure S1.** Relationship between changes in serum uric acid levels and changes in urinary protein and albumin at week 24 from baseline. **Table S1.** Patient background in each of the 5 CKD stage groups. **Table S2.** Serum uric acid levels. **Table S3.** Indicators related to renal function. **Table S4.** Uric acid clearance-to-creatinine clearance ratio (C_UA_/C_Cr_). **Table S5.** Other safety indicators (liver function tests and urinary pH). STROBE Checklist.

## Footnotes

Supplementary File (PDF)
**Table S1.** Patient background in each of the 5 CKD stage groups. **Table S2.** Serum uric acid levels. **Table S3.** Indicators related to renal function. **Table S4.** Uric acid clearance-to-creatinine clearance ratio (C_UA_/C_Cr_). **Table S5.** Other safety indicators (liver function tests and urinary pH). **Figure S1.** Relationship between changes in serum uric acid levels and changes in urinary protein and albumin at week 24 from baseline. STROBE Checklist.
