# Supplementary material for: Dotinurad Treatment for Patients With Hyperuricemia Complicating CKD
Source: Kidney Int Rep. 2025 Apr 3;10(6):1711–20. doi: 10.1016/j.ekir.2025.03.047 (PMC12232968; doi:10.1016/j.ekir.2025.03.047)
Supplement: Supplementary File (PDF) — Figure S1. Relationship between changes in serum uric acid levels and changes in urinary protein and albumin at week 24 from baseline. Table S1. Patient background in each of the 5 CKD stage groups. Table S2. Serum uric acid levels. Table S3. Indicators related to renal function. Table S4. Uric acid clearance-to-creatinine clearance ratio (CUA/CCr). Table S5. Other safety indicators (liver function tests and urinary pH). STROBE Checklist. [file mmc1.pdf]

Supplemental Material

Table of contents

1. Supplemental Table 1. Patient background in each of the five CKD stage groups

2. Supplemental Table 2. Serum uric acid levels

3. Supplemental Table 3. Indicators related to renal function

4. Supplemental Table 4. C_{UA}/C_{Cr}

5. Supplemental Table 5. Other safety indicators (liver function tests and urinary pH)

6. Supplemental Figure 1. Relationship between changes in serum uric acid levels and changes in urinary protein and albumin at Week 24 from baseline

7. STROBE Checklist

Supplemental Table 1 - Patient background in each of the five CKD stage groups

Group		G1 (*n*=10)	G2 (*n*=40)	G3a (*n*=16)	G3b (*n*=19)	G4 (*n*=13)
Sex	Male	9 (90)	38 (95)	14 (88)	17 (90)	8 (62)
Age (years)	Mean (SD)	36 (10)	55 (13)	64 (13)	65 (14)	67 (14)
	20–59	10 (100)	26 (65)	6 (38)	5 (26)	4 (31)
	≥60	0	14 (35)	10 (63)	14 (74)	9 (69)
BMI (kg/m^2)	Mean (SD)	33.8 (4.2)	27.0 (3.8)	24.7 (3.5)	26.3 (4.5)	25.4 (4.8)
	18.5 to <25	0	15 (38)	11 (69)	9 (47)	7 (54)
	25 to <30	1 (10)	15 (38)	4 (25)	6 (32)	4 (31)
	≥30	9 (90)	10 (25)	1 (6)	4 (21)	2 (15)
Duration of hyperuricemia (years)	Mean (SD)	2.5 (4.4)	3.0 (4.8)	3.2 (4.6)	5.4 (7.5)	3.6 (8.6)
	<1	6 (60)	19 (48)	8 (50)	10 (53)	8 (62)
	1–9	2 (20)	17 (43)	5 (31)	5 (26)	4 (31)
	≥10	2 (20)	4 (10)	3 (19)	4 (21)	1 (8)
Serum UA level (mg/dL)	Mean (SD)	9.0 (0.7)	8.5 (0.9)	8.9 (0.9)	8.9 (0.7)	9.1 (1.2)
	Min, max	8.3, 10.1	4.8, 10.0	7.7, 11.5	8.0, 10.1	8.1, 12.6
eGFR (mL/min/1.73m^2)	Mean (SD)	101.1 (12.2)	72.4 (7.8)	51.7 (3.7)	37 (4.7)	22.6 (4.5)
	Min, max	91.2, 130.3	60.3, 88.3	46.3, 58.5	31.0, 44.8	15.8, 28.6
Urine protein/creatinine ratio (g/gCr)	Mean (SD)	0.108 (0.122)	0.153 (0.334)	0.553 (0.767)	0.618 (0.881)	1.976 (2.068)
	Min, max	0.02, 0.42	0.01, 1.84	0.02, 2.18	0.03, 3.51	0.06, 5.39

Urine albumin/creatinine ratio (mg/gCr)	Mean (SD)	44.4 (82.4)	91.5 (265.7)	381.0 (523.1)	524.4 (689.1)	1253.0 (1447.9)
	Min, max	3, 270	3, 1248	8, 1389	4, 2361	9, 3230
History of gout	Yes	0	10 (25)	3 (19)	3 (16)	1 (8)
CKD[a]	Yes	0	0	16 (100)	19 (100)	13 (100)
Causal disease	Diabetic nephropathy			3 (19)	6 (32)	5 (39)
	Nephrosclerosis			8 (50)	7 (37)	3 (23)
	IgA nephropathy			2 (13)	3 (16)	0
	Membranous nephropathy			1 (6)	0	0
	Focal glomerulo-sclerosis			0	1 (5)	1 (8)
	Polycystic kidney disease			0	0	2 (15)
	Unknown cause			2 (13)	0	1 (8)
	Others			0	2 (11)	1 (8)
	Malignant neoplasm of renal pelvis				1 (5)	0
	Nephrectomy				0	1 (8)
	Toxic nephropathy				1 (5)	0
Comorbidities	Yes	10 (100)	33 (83)	15 (94)	17 (90)	13 (100)
	Diabetes	7 (70)	16 (40)	8 (50)	8 (42)	8 (62)
	Dyslipidemia	7 (70)	21 (53)	11 (69)	12 (63)	9 (69)
	Hypertension	4 (40)	23 (58)	13 (81)	14 (74)	10 (77)
	Stroke or CAD	0	5 (13)	2 (13)	4 (21)	4 (31)
	PAD	0	0	0	0	0

Concomitant medications	Thiazides	1 (10)	3 (7)	0 (0)	1 (5)	1 (7)
	Diuretics except thiazides	0 (0)	0 (0)	1 (6)	1 (5)	2 (15)
	ARBs	0 (0)	2 (5)	0 (0)	0 (0)	2 (15)
	Antihypertensives except diuretics and ARBs	4 (40)	20 (50)	9 (56)	8 (42)	8 (61)
	Insulin	0 (0)	1 (2)	2 (12)	1 (5)	5 (38)
	SGLT2-inhibitors	6 (60)	9 (23)	6 (38)	6 (32)	4 (31)

Values are *n* (%) unless indicated otherwise.

[a]Defined as eGFR <60 mL/min/1.73 m^2.

Abbreviations: ARB, angiotensin II receptor blockers; BMI, body mass index; CAD, coronary artery disease; CKD, chronic kidney disease; eGFR, estimated glomerular filtration rate; IgA, immunoglobulin A; PAD, peripheral artery disease; SD, standard deviation; SGLT2, sodium-glucose cotransporter 2, UA, uric acid.

Supplemental Table 2 - Serum uric acid levels

Group	*n*	Serum UA level, mg/dL [mean (SD)]					% Decrease in serum UA level from baseline, % [LS mean (95% CI)]					Achieved target UA level at Week 24	
		Week 0 (baseline)	Week 4	Week 8	Week 12	Week 24	Week 4	Week 8	Week 12	Week 24	Inter-group difference	*n* (%)	Difference in proportion, % [mean (95% CI)]
G1	10	9.0 (0.7)	7.5 (1.4)	6.3 (1.3)	4.9 (1.5)	4.7 (0.7)	17.3 (11.2 to 23.5)	30.8 (22.7 to 38.9)	47.1 (37.6 to 56.6)	48.9 (41.1 to 56.7)	ref.	10 (100)	
G2	40	8.5 (0.9)	6.6 (1.1)	5.7 (1.0)	4.5 (1.0)	4.5 (1.0)	22.2 (19.1 to 25.3)	32.6 (28.5 to 36.7)	46.4 (41.6 to 51.2)	46.8 (42.8 to 50.8)	−2.1 (−10.8 to 6.7)	36 (92)	
G3a	16	8.9 (0.9)	7.5 (0.9)	6.5 (1.4)	5.0 (1.4)	4.9 (1.4)	15.7 (10.9 to 20.6)	27.3 (20.9 to 33.7)	43.6 (36.0 to 51.1)	45.4 (39.3 to 51.6)	−3.4 (−13.3 to 6.5)	11 (69)	
G3b	19	8.9 (0.7)	7.2 (1.1)	6.5 (1.4)	5.2 (1.3)	4.8 (1.1)	18.1 (13.7 to 22.5)	26.1 (20.3 to 31.9)	39.9 (33.2 to 46.6)	45.5 (39.8 to 51.2)	−3.4 (−13.0 to 6.2)	16 (89)	
G4	13	9.1 (1.2)	7.9 (1.9)	7.5 (2.3)	6.6 (2.6)	6.0 (2.1)	13.7 (8.3 to 19.1)	18.8 (11.7 to 25.9)	29.4 (21.1 to 37.8)	35.7 (28.8 to 42.5)	−13.2 (−23.5 to −2.9)	7 (54)	
G1/G2	50	8.6 (0.9)	6.8 (1.2)	5.8 (1.1)	4.6 (1.1)	4.5 (1.0)	21.2 (18.4 to 24.0)	32.2 (28.5 to 35.9)	46.5 (42.1 to 50.9)	47.2 (43.6 to 50.8)	ref.	46 (94)	ref.
G3/G4	48	8.9 (0.9)	7.5 (1.3)	6.8 (1.7)	5.5 (1.9)	5.1 (1.6)	16.2 (13.3 to 19.0)	24.6 (20.8 to 28.3)	38.3 (33.9 to 42.7)	42.8 (39.1 to 46.4)	−4.4[a] (−9.5 to 0.7)	34 (72)	−21.5[b] (−37.0 to −6.6)

[a]Inter-group difference: *P*=0.0912 for null hypothesis "difference = 0".

[b]Inter-group difference: *P*=0.0058 for null hypothesis "difference=0".

Abbreviations: CI, confidence interval; LS, least squares; ref., reference; SD, standard deviation; UA, uric acid.

Supplemental Table 3 - Indicators related to renal function

Group	eGFR, mL/min/1.73 m^2					Urine Protein/Cr, g/gCr				Urine Albumin/Cr, mg/gCr			
	n	Week 0 (baseline)	Week 24	Change [LS mean (95% CI)]		*n*	Week 0 (baseline)	Week 24	Change	*n*	Week 0 (baseline)	Week 24	Change
				Value	Inter-group difference								
G1	10	101 (12)	109 (20)	5.8 (−7.4 to 18.9)	ref.	10	0.1 (0.1)	0.1 (0.0)	0.0 (0.1)	10	44.4 (82.4)	25.0 (33.3)	−25.6 (65.8)
G2	40	72 (8)	74 (13)	0.6 (−4.3 to 5.4)	−5.2 (−15.5 to 5.2)	40	0.2 (0.3)	0.2 (0.4)	0.0 (0.1)	30	91.5 (265.7)	123.8 (363.0)	21.6 (93.8)
G3a	16	52 (4)	52 (8)	0.7 (−4.3 to 5.7)	−5.1 (−20.7 to 10.6)	16	0.6 (0.8)	0.3 (0.3)	−0.3 (0.7)	11	381.0 (523.1)	171.4 (179.4)	−234.1 (455.8)
G3b	19	37 (5)	40 (8)	3.5 (−4.0 to 11.0)	−2.2 (−21.5 to 17.1)	19	0.6 (0.9)	0.8 (1.1)	0.2 (0.7)	11	524.4 (689.1)	448.7 (576.7)	−9.4 (268.9)
G4	13	23 (5)	25 (8)	3.5 (−7.7 to 14.8)	−2.2 (−25.4 to 21.0)	13	2.0 (2.1)	1.7 (1.9)	−0.3 (1.5)	8	1253.0 (1447.9)	1301.1 (1788.2)	48.0 (1095.0)
G1/G2	50	78 (15)	81 (20)	1.0 (−2.9 to 4.9)	ref.	50	0.1 (0.3)	0.2 (0.4)	0.0 (0.1)	40	79.8 (233.4)	100.5 (319.2)	10.2 (89.3)
G3/G4	48	38 (12)	40 (13)	3.2 (−0.8 to 7.2)	2.2 (−4.7 to 9.1)	48	1.0 (1.4)	0.9 (1.3)	−0.1 (1.0)	30	666.1 (947.4)	574.3 (1054.0)	−75.6 (657.9)

Values are mean (SD) unless indicated otherwise.

Abbreviations: Cr, creatinine; CI, confidence interval; eGFR, estimated glomerular filtration rate; LS, least squares; SD, standard deviation.

Supplemental Table 4 - C_{UA}/C_{Cr}

Group	*n*	C_{UA}/C_{Cr}, %					Change (Increase) in C_{UA}/C_{Cr} from baseline, %			
		Week 0 (baseline)	Week 4	Week 8	Week 12	Week 24	Week 4	Week 8	Week 12	Week 24
G1	10	5.7 (1.9)	5.4 (2.6)	6.8 (3.6)	11.4 (7.2)	8.7 (3.5)	−0.3 (3.2)	1.2 (3.8)	5.7 (6.7)	3.0 (3.3)
G2	40	4.9 (2.1)	7.1 (3.5)	9.2 (5.2)	12.6 (6.7)	11.8 (6.0)	2.2 (2.8)	4.4 (4.9)	7.9 (6.7)	7.0 (5.8)
G3a	16	6.9 (2.5)	8.3 (3.0)	10.6 (4.4)	14.7 (7.1)	14.0 (5.8)	1.4 (3.6)	3.7 (5.4)	7.8 (8.0)	7.2 (5.9)
G3b	19	6.0 (1.8)	7.7 (3.2)	9.7 (3.6)	12.2 (5.1)	13.9 (4.4)	1.5 (2.4)	3.7 (3.6)	5.7 (4.9)	7.9 (4.8)
G4	13	9.5 (3.7)	10.5 (3.5)	12.6 (4.0)	14.8 (4.9)	17.5 (7.1)	1.1 (4.2)	3.3 (4.7)	5.1 (6.0)	8.2 (7.6)

Values are mean (SD).

Abbreviations: C_{UA}/C_{Cr}, uric acid clearance/creatinine clearance ratio; SD, standard deviation.

Supplemental Table 5 - Other safety indicators (liver function tests and urinary pH)

Group	AST (IU/L)				ALT (IU/L)				γ-GTP (IU/L)				Urinary pH				Casual blood glucose (mg/dL)				HbA1c (%)			
	n	Week 0 (base-line)	Week 24	Change	n	Week 0 (base-line)	Week 24	Change	n	Week 0 (base-line)	Week 24	Change	n	Week 0 (base-line)	Week 24	Change	n	Week 0 (base-line)	Week 24	Change	n	Week 0 (base-line)	Week 24	Change
G1	10	31.4 (14.4)	39.7 (24.8)	8.3 (15.8)	10	48.9 (15.6)	62.9 (38.9)	14.0 (30.6)	10	71.1 (39.7)	84.9 (64.2)	13.8 (37.9)	10	6.5 (1.0)	6.1 (0.8)	−0.4 (1.1)	10	130.8 (24.8)	149.1 (51.7)	18.3 (46.7)	10	7.0 (1.6)	7.5 (1.7)	0.5 (1.1)
G2	40	25.5 (8.1)	25.4 (9.4)	0.3 (6.0)	40	32.1 (16.9)	30.0 (15.5)	−0.4 (12.2)	40	63.8 (55.4)	64.0 (53.9)	−0.8 (23.2)	40	6.0 (0.7)	6.0 (0.6)	0.0 (0.8)	40	128.5 (45.8)	137.7 (45.6)	10.2 (49.7)	33	6.2 (0.9)	6.1 (1.0)	0.0 (0.4)
G3a	16	23.3 (8.6)	19.7 (4.9)	−3.6 (9.6)	16	20.2 (10.4)	18.4 (5.5)	−1.8 (8.9)	16	38.6 (24.5)	42.7 (31.3)	4.1 (12.7)	16	6.1 (0.8)	6.0 (0.6)	−0.1 (0.8)	16	121.5 (38.2)	138.0 (48.8)	16.5 (30.8)	15	6.7 (1.8)	6.5 (1.5)	−0.3 (0.7)
G3b	21	25.3 (15.6)	20.7 (7.6)	−5.8 (13.9)	21	22.2 (11.8)	18.2 (5.8)	−5.6 (9.8)	21	77.8 (135.3)	54.3 (64.1)	−29.4 (101.9)	21	5.5 (0.5)	5.8 (0.5)	0.2 (0.6)	21	126.7 (32.5)	120.5 (21.3)	−5.7 (25.8)	14	6.9 (1.4)	6.2 (0.6)	−0.6 (1.3)
G4	13	22.6 (11.1)	18.5 (5.2)	−4.1 (11.2)	13	18.8 (13.3)	14.2 (6.6)	−4.5 (14.0)	13	34.6 (28.2)	35.2 (48.5)	0.5 (25.8)	13	5.9 (0.9)	6.4 (0.9)	0.5 (0.6)	13	130.3 (56.4)	139.0 (43.1)	8.7 (43.9)	8	7.0 (1.1)	6.6 (1.0)	−0.1 (0.6)

Values are mean (SD).

Abbreviations: ALT, alanine aminotransferase; AST, aspartate aminotransferase; γ-GTP, γ-glutamyl transpeptidase; HbA1c, glycated hemoglobin; SD, standard deviation.

Supplemental Figure 1 - Relationship between changes in serum uric acid levels and changes in urinary protein and albumin at Week 24 from baseline

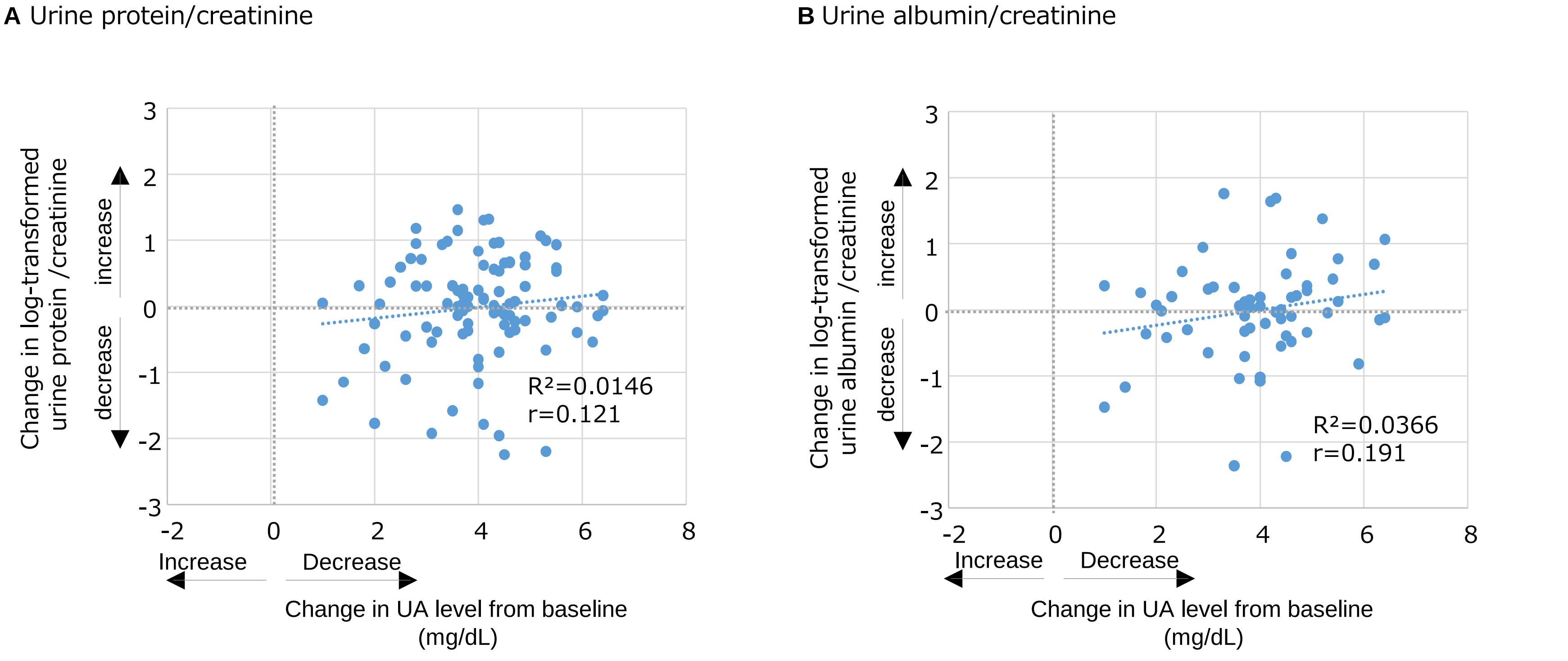

Vertical axis: log(Urine protein /creatinine (at Week 24)) - log(Urine protein /creatinine (baseline))

Vertical axis: log(Urine albumin /creatinine (at Week 24)) - log(Urine albumin /creatinine (baseline))

STROBE Statement—checklist of items that should be included in reports of observational studies

	Item No	Recommendation	Page No
**Title and abstract**	1	(*a*) Indicate the study's design with a commonly used term in the title or the abstract	3
		(*b*) Provide in the abstract an informative and balanced summary of what was done and what was found	3
**Introduction**			
Background/rationale	2	Explain the scientific background and rationale for the investigation being reported	4, 5
Objectives	3	State specific objectives, including any prespecified hypotheses	5
**Methods**			
Study design	4	Present key elements of study design early in the paper	6
Setting	5	Describe the setting, locations, and relevant dates, including periods of recruitment, exposure, follow-up, and data collection	6, 7
Participants	6	(*a*) *Cohort study*—Give the eligibility criteria, and the sources and methods of selection of participants. Describe methods of follow-up<br>*Case-control study*—Give the eligibility criteria, and the sources and methods of case ascertainment and control selection. Give the rationale for the choice of cases and controls<br>*Cross-sectional study*—Give the eligibility criteria, and the sources and methods of selection of participants	6
		(*b*) *Cohort study*—For matched studies, give matching criteria and number of exposed and unexposed<br>*Case-control study*—For matched studies, give matching criteria and the number of controls per case	NA
Variables	7	Clearly define all outcomes, exposures, predictors, potential confounders, and effect modifiers. Give diagnostic criteria, if applicable	7
Data sources/ measurement	8*	For each variable of interest, give sources of data and details of methods of assessment (measurement). Describe comparability of assessment methods if there is more than one group	7
Bias	9	Describe any efforts to address potential sources of bias	8
Study size	10	Explain how the study size was arrived at	8
Quantitative variables	11	Explain how quantitative variables were handled in the analyses. If applicable, describe which groupings were chosen and why	8
Statistical methods	12	(*a*) Describe all statistical methods, including those used to control for confounding	8, 9
		(*b*) Describe any methods used to examine subgroups and interactions	NA
		(*c*) Explain how missing data were addressed	8
		(*d*) *Cohort study*—If applicable, explain how loss to follow-up was addressed<br>*Case-control study*—If applicable, explain how matching of cases and controls was addressed<br>*Cross-sectional study*—If applicable, describe analytical methods taking account of sampling strategy	
		(*e*) Describe any sensitivity analyses	NA

Continued on next page

**Results**			
Participants	13*	(a) Report numbers of individuals at each stage of study—eg numbers potentially eligible, examined for eligibility, confirmed eligible, included in the study, completing follow-up, and analysed	9, Figure 1
		(b) Give reasons for non-participation at each stage	9, Figure 1
		(c) Consider use of a flow diagram	Figure 1
Descriptive data	14*	(a) Give characteristics of study participants (eg demographic, clinical, social) and information on exposures and potential confounders	9, Tables 1, S1.
		(b) Indicate number of participants with missing data for each variable of interest	10
		(c) *Cohort study*—Summarise follow-up time (eg, average and total amount)	
Outcome data	15*	*Cohort study*—Report numbers of outcome events or summary measures over time	9–11, Figures 3-6, Tables 2 and S2-S5
		Case-control study—Report numbers in each exposure category, or summary measures of exposure	
		Cross-sectional study—Report numbers of outcome events or summary measures	
Main results	16	(*a*) Give unadjusted estimates and, if applicable, confounder-adjusted estimates and their precision (eg, 95% confidence interval). Make clear which confounders were adjusted for and why they were included	9–11, Figures 3-6, Tables 2 and S2-S5
		(*b*) Report category boundaries when continuous variables were categorized	
		(*c*) If relevant, consider translating estimates of relative risk into absolute risk for a meaningful time period	
Other analyses	17	Report other analyses done—eg analyses of subgroups and interactions, and sensitivity analyses	NA
**Discussion**			
Key results	18	Summarise key results with reference to study objectives	11–12
Limitations	19	Discuss limitations of the study, taking into account sources of potential bias or imprecision. Discuss both direction and magnitude of any potential bias	13
Interpretation	20	Give a cautious overall interpretation of results considering objectives, limitations, multiplicity of analyses, results from similar studies, and other relevant evidence	13
Generalisability	21	Discuss the generalisability (external validity) of the study results	13
**Other information**			
Funding	22	Give the source of funding and the role of the funders for the present study and, if applicable, for the original study on which the present article is based	14

*Give information separately for cases and controls in case-control studies and, if applicable, for exposed and unexposed groups in cohort and cross-sectional studies.

Note: An Explanation and Elaboration article discusses each checklist item and gives methodological background and published examples of transparent reporting. The STROBE checklist is best used in conjunction with this article (freely available on the Web sites of PLoS Medicine at http://www.plosmedicine.org/, Annals of Internal Medicine at http://www.annals.org/, and Epidemiology at http://www.epidem.com/). Information on the STROBE Initiative is available at www.strobe-statement.org.
